# Bridging Physical and Mental Health in Child and Adolescent Mental Health Services (CAMHS): A Simulation Based Quality Improvement Project (QIP) for Medical Students

**DOI:** 10.1192/bjo.2026.11301

**Published:** 2026-06-30

**Authors:** Thakshayene Mahenthran, Sabrina Alam, Michael Groszmann, Yasmin Baki, Camilla Sen

**Affiliations:** University College London Hospital, London, United Kingdom

## Abstract

**Aims:**

Year 5 medical students on paediatric placements at University College London Hospitals (UCLH) reported limited and inconsistent exposure to Child and AdolescentMental Health Services (CAMHS), with concerns that existing placements were not always relevant or applicable to undergraduate learning needs. This quality improvement project (QIP) aimed to improve Year 5 medical students’ self-reported confidence in assessing and communicating with young people in mental health settings through a structured simulation session integrating physical and mental health presentations.

**Methods:**

The problem was identified through placement feedback surveys, discussions with student representatives, and analysis with undergraduate CAMHS and paediatric leads alongside a review of the Year 5 curriculum. In response, asimulation-based teaching afternoon was co-developed with CAMHS liaison psychiatrists, paediatric doctors, CAMHS liaison clinical nurse specialists, and a medical student representative to ensure educational relevance and clinical authenticity.

Four simulation scenarios were co-designed to reflect common CAMHS presentations while integrating physical health considerations, psychosocial assessment, and communication skills. Scenarios were selected based on evaluation of existing teaching sessions to avoid topic repetition and to cover areas of the curriculum that were not otherwise addressed. Role-players were volunteering doctors, briefed on each scenario and given time to review the scripts, ensuring the simulations were authentic, structured, and cost-effective. The session was delivered to a pilot group of twelve Year 5 medical students during their paediatric and CAMHS placements. Each scenario was followed by a structured debrief focusing on communication with adolescents, holistic assessment, and integration of physical and mental health care. Students completed anonymised pre-and post-session questionnaires using 5-point Likert scales and free-text feedback.

**Results:**

Pre-simulation questionnaires demonstrated low to moderate confidence. Only 50%of students felt confident communicating with adolescent patients, 25% felt confident managing consultations involving both physical and mental health issues, 25–33% felt confident using a structured psychosocial approach or asking sensitive questions, and 33–50% felt prepared for OSCEs.

Post-simulation feedback showed marked improvement across all domains. 92% of students reported increased confidence communicating with adolescent patients, 83% felt confident building rapport in time-limited consultations, 83% felt confident exploring psychosocial factors, 92% felt confident using structured psychosocial approaches, and 83% felt prepared for OSCEs involving mental health components. Students also rated the simulation as a highly useful method for developing communication skills.

Qualitative analysis identified two key themes. First, students valued practising difficult and sensitive conversations in a safe environment (“It was a good chance to practise how to phrase things in difficult scenarios”). Second, they highlighted the benefit of realistic scenarios integrating physical and mental health, enhancing understanding of holistic assessment (“Seeing how different physical health scenarios may play into psych”).

**Conclusion:**

This QIP successfully co-developed and piloted a novel simulation-based teaching intervention addressing gaps in undergraduate CAMHS education. Pre-and post-session evaluation suggests it is effective in improving medical students’ confidence and perceived preparedness for working with young people in mental health settings. The simulation will be embedded into future CAMHS rotations at UCLH, with iterative refinement guided by learner feedback. This project demonstrates how co-designed simulation can enhance undergraduate psychiatric education and support integrated physical and mental health learning.

